# 1674. 10 Year p-OPAT Experience In A Tertiary Pediatric Center In Singapore

**DOI:** 10.1093/ofid/ofad500.1507

**Published:** 2023-11-27

**Authors:** Rie Aoyama, Si Min Chan, Olivla Leow, Samantha Ang, Belinda Quek

**Affiliations:** Khoo Teck Puat National University Children's Medical Institute, Singapore, Singapore; Khoo Teck Puat National University Children's Medical Institute, Singapore, Singapore; Khoo Teck Puat National University Children's Medical Institute, Singapore, Singapore; Khoo Teck Puat National University Children's Medical Institute, Singapore, Singapore; Khoo Teck Puat National University Children's Medical Institute, Singapore, Singapore

## Abstract

**Background:**

Outpatient parental antibiotic therapy is safe and effective. Our institution is one of two tertiary public hospital pediatric centers in Singapore. We aim to describe characteristics and outcomes of pediatric OPAT (p-OPAT).

**Methods:**

We retrospectively reviewed p-OPAT patients from May 2013-Dec 2022. Clinically stable inpatients were referred to the pediatric infectious disease team for assessment, caregiver training, and weekly review. Two main delivery models were used; daily bolus/short infusion at an outpatient unit, and home self-administration via elastomeric device. Electronic medical records were analyzed for demographics, clinical and microbiological data, treatment, and outcomes. Institutional review board approval and written informed consent were waived.

**Results:**

219 patients completed 249 p-OPAT episodes; 46.6% female, 53.3% male. Median age was 8 years 5 months (range 11 days-29 years). Patient ethnicities were 53.9% Chinese, 12.3% Indian, 20.5% Malay, 13.3% others.

53/219 (24.2%) patients were immunocompromised; three had primary immunodeficiency, 50 had acquired immunodeficiency. 89/219 (40.6%) patients had chronic illness.

176/249 (70.7%) episodes had only one pathogen identified (Tab 1). Pathogens were gram positive (21.7%), gram negative (38.2%), fungal (5.2%), mycobacterial (5.6%) (Tab 2). 18.9% episodes were culture-negative, 1.6% episodes had no cultures. Infection sites varied considerably; osteoarticular infection +/- bacteremia were most common (Tab 3). Ceftriaxone was the most frequent antimicrobial (105, 42.3% episodes). Peripherally inserted central catheter was most commonly used (138, 55.4% episodes).

Complete success, partial success, and failure were seen in 174 (69.9%), 43 (17.3%) and 32 (12.8%) episodes respectively. Main adverse events (AE) were vascular site dermatitis, infection, and line dislodgement. 235 (94.4%) episodes achieved infection cure. Mean p-OPAT duration was 16.9 days (range 1-177 days), saving 4208 admission bed days.

**Figure:**
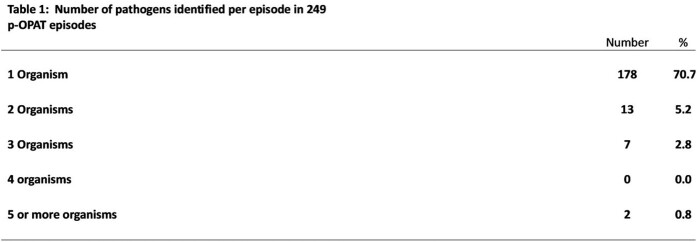

**Figure:**
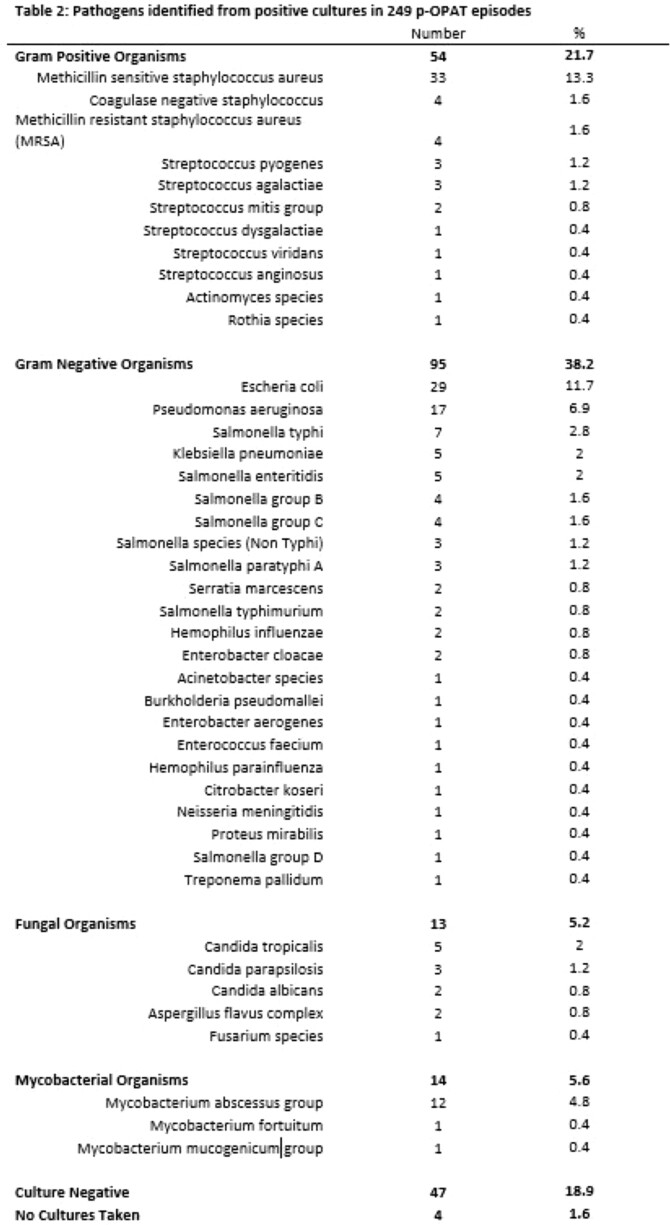

**Figure:**
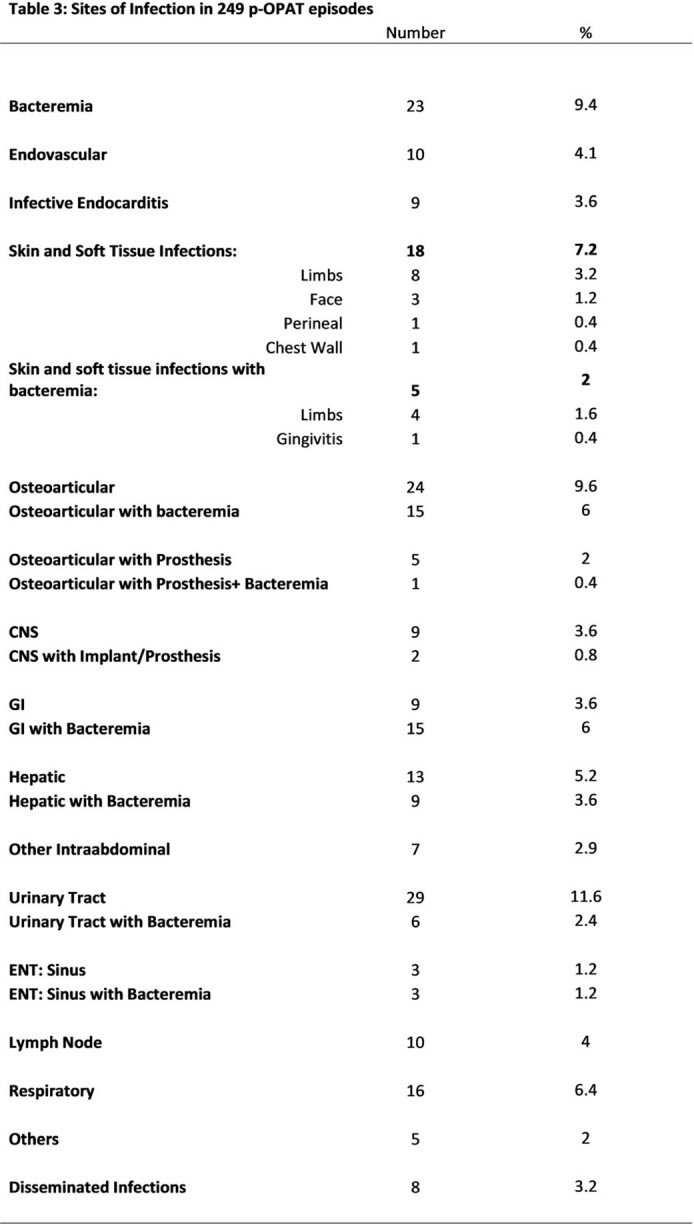

**Conclusion:**

We describe one of the largest series of children receiving p-OPAT over 10 years and report good outcomes for a broad spectrum of infections. Vascular access issues were common AEs despite preventive measures.

**Disclosures:**

**All Authors**: No reported disclosures

